# Investigation of the Effect of Asprosin Level on Fertility Success and Oxidative Stress in Simmental Cows

**DOI:** 10.1111/rda.70022

**Published:** 2025-02-25

**Authors:** Rahmi Köse, Serkan Ali Akarsu, Serdal Kurt, Hakan Serin, Mehmet Akif Aydın, Ahmet Karahanlı, Gamze Uçak, Ahmet Yörü

**Affiliations:** ^1^ Department of Reproduction and Artificial Insemination, Faculty of Veterinary Medicine Ataturk University Erzurum Turkey; ^2^ Department of Veterinary Elbistan Vocational School, Kahramanmaras Istiklal University, Kahramanmaras Turkey; ^3^ Department of Biostatistics, Faculty of Veterinary Medicine Selçuk University Konya Turkey; ^4^ Department of Reproduction and Artificial Insemination, Faculty of Veterinary Medicine Kafkas University Kars Turkey

**Keywords:** artificial insemination, asprosin, cow, oxidative stress, pregnancy

## Abstract

Asprosin, a protein hormone that regulates hunger and glucose homeostasis, is produced by white adipose tissue. The aim of this study was to investigate the effect of asprosin level on fertility and oxidative stress parameters in Simmental cows. In the study, 44 Simmental multiparous cows were used. PRID was used to synchronise the animals. Blood samples were obtained from the cattle on the day of artificial insemination, and the amount of asprosin was measured. Animals were divided into two groups: a low asprosin (LA) group and a high asprosin (HA) group. On day 25, blood samples were taken, and pregnancy‐associated glycoprotein (PAG2), total antioxidant level (TAS) and total oxidant level (TOS) were measured using commercial kits. Ultrasonography was used to diagnose pregnancy on day 45. The TAS level at day 25 was higher in the HA group than the TAS level at the beginning of the study (*p* = 0.007). The TOS level at the beginning of the study was higher in the HA group than in the LA group (*p* = 0.017). The TOS level of cows in the LA group on day 25 was significantly higher than the TOS level at the beginning of the study (*p* = 0.028). In conclusion, asprosin levels in cows affected oxidative stress parameters. According to receiver operating characteristic curve (ROC) analysis, there was a strong correlation between TOS and asprosin, and it was concluded that cows with HA values may be exposed to oxidative stress.

## 1 Introduction

Cows undergo rapid metabolic and immunological changes, especially during the first 3–4 weeks before and after parturition (Bühler et al. 2018; Sundrum 2015). During this process, cows experience negative energy balance (NEB) because of decreased appetite and increased energy needs due to the sudden start of milk production (Bisinotto et al. 2015). The energy needed as a result of NEB is met from non‐esterified fatty acids (NEFA) released by the mobilisation of adipose tissues (Li et al. 2022). On the other hand, various bioactive substances in the form of proteins called adipocytokines are released during the metabolism of adipose tissues (Aktaş et al. 2013; Demirci and Gün 2019; Farrag et al. 2023). Adipocytokines have important roles in the immunological and glucose system. In recent years, a mediator called asprosin has become a subject of curiosity due to its effects on the immune system, insulin release and energy metabolism (Farrag et al. 2023; Mazur‐Bialy 2021). Asprosin is secreted in a state of hunger. It is reported that it crosses the blood–brain barrier and stimulates the hypothalamic nutrition centre, increasing appetite, and then its level decreases again with feeding (Duerrschmid et al. 2017). It is also known that asprosin maintains glucose homeostasis during fasting and obesity by promoting hepatic glucose production (Li et al. 2019). Increasing the metabolism of adipose tissues increases the production of reactive oxygen species (ROS) known as oxidants. In other words, increased NEFA oxidation increases the production of oxygen radicals in the liver. Thus, the use of antioxidant substances increases. If oxidant substances exceed the capacity of antioxidant substances, this results in oxidative stress (Li et al. 2022; Song et al. 2014). In case of oxidative stress, structural deteriorations occur in macromolecules of cells such as DNA, lipids and proteins (Li et al. 2022; Rolo et al. 2012; Sordillo and Aitken 2009). Gametes in particular are sensitive to oxidative damage. It is known that in case of oxidative stress, the fertilisation rate decreases, ovarian activities are impaired, implantation problems and embryonic death cases increase (Wang et al. 2021). For these reasons, it is understood that the antioxidant level must be high and the oxidant level must be low for a successful fertility process in cows. Current research highlights the relationship between asprosin and fertility. It is reported that asprosin improves the decrease in fertility potential due to age and obesity by increasing sperm motility (Mazur‐Bialy 2021). Insulin resistance cases are associated with high asprosin (HA) levels. It is also known that changes in energy metabolism and insulin secretion function as a result of NEB seriously negatively affect fertility success in cows (Baruselli et al. 2016; Civiero et al. 2021).

The aim of this study was to investigate the impact of asprosin levels on fertility and oxidative stress parameters in Simmental cows. According to the literature review, no study was able to fully explain the role of asprosin in fertilisation. Asprosin levels are positively correlated with insulin resistance, body mass index and free androgen index.

## 2 Material and Method

### 2.1 Ethical Approval and Animals

The presented study was approved by the decision numbered 167 of the Atatürk University Animal Experiments Local Ethics Committee in the session numbered 2023/11, dated 28 September 2023. The presented study was conducted on 44 Simmental breed multiparous cows at Atatürk University Application and Research Farm, where professional animal husbandry procedures and preventive medicine services are provided. The animals were selected from cows that were fed according to their physiological and individual needs, were of similar age and lactation period, did not have any health problems, were raised in free‐stall barns, and were milked twice a day.

### 2.2 Grouping

A total of 44 cows used in the study were divided into two groups as low asprosin level (LA group; *n*: 22) and HA level (HA group; *n*: 22) according to the asprosin level at the beginning of the study (day 0). Asprosin levels were measured from blood samples taken on day 0 and day 25 ± 2 of the study, and the cows with the lowest 22 measurements were recorded in the LA group using statistical measurement methods in measurements on day 0. The other 22 cows, which had higher asprosin values than the LA group, were recorded in the HA group. Cows in the LA and HA groups were subjected to the oestrus synchronisation programme named PRID, and the insemination day was considered as day 0 of the study.

### 2.3 Blood Collection

In order to determine the asprosin, pregnancy‐associated glycoprotein (PAG2), total antioxidant level (TAS) and total oxidant level (TOS) values, blood samples were collected from the coccygeal veins of the cows into anticoagulant‐free tubes (10 mL) on day 0 and day 25 ± 2 of the study. The blood samples taken were sent to the laboratory and centrifuged at 1500 × *g* at +4°C for 15 min, and serum samples were harvested. The obtained serum samples were stored at −20°C until the day of analysis.

### 2.4 Biochemical Analysis

#### 2.4.1 TAS and TOS Analysis

TAS and TOS levels were measured using commercially available kits (Relassay, Turkey). The inter‐and intra‐assay coefficients of TAS and TOS kits were 2.8%; 3.3% and 3.2%; 3.9%, respectively. Their measurement technique was the colorimetric method. The novel automated method is based on the bleaching of the characteristic colour of a more stable ABTS (2,2′‐Azino‐bis (3‐ethylbenzothiazoline‐6‐sulfonic acid)) radical cation by antioxidants. The assay has excellent precision values, which are lower than 3%. The results were expressed as mmol Trolox equivalent/L (Erel 2004; Eşki et al. 2021).

TOS levels were measured using commercially available kits (Relassay, Turkey). In the new method, oxidants present in the sample oxidised the ferrous ion‐o‐dianisidine complex to ferric ion. The oxidation reaction was enhanced by glycerol molecules abundantly present in the reaction medium. The ferric ion produced a coloured complex with xylenol orange in an acidic medium. The colour intensity, which could be measured spectrophotometrically, was related to the total amount of oxidant molecules present in the sample. The assay was calibrated with hydrogen peroxide, and the results were expressed in terms of micromolar hydrogen peroxide equivalent per litre (μmol H_2_O_2_ equivalent/L) (Erel 2005).

#### 2.4.2 Bovine Pregnancy Associated Glycoprotein 2

PAG2 level was measured by the ELISA method using a commercial kit (BTlab, Cat. No. E2335Bo). The plate has been pre‐coated with Bovine PAG2 antibody. PAG2 present in the sample is added and binds to antibodies coated on the wells. Then, biotinylated Bovine PAG2 antibody is added and binds to PAG2 in the sample. Next, streptavidin‐HRP is added and binds to the Biotinylated PAG2 antibody. After incubation, unbound streptavidin‐HRP is washed away during a washing step. Substrate solution is then added and colour develops in proportion to the amount of Bovine PAG2. The reaction is terminated by the addition of acidic stop solution, and absorbance is measured at 450 nm. The detection limit of PAG2 was between 0.1 and 40 ng/mL, and the functional sensitivity is 0.051 ng/mL.

#### 2.4.3 Asprosin Level

Asprosin level was measured by the ELISA method using a commercial kit (BTlab, Cat. No. E0486Bo). The plate has been pre‐coated with Bovine asprosin antibody. Asprosin present in the sample is added and binds to antibodies coated on the wells. Then biotinylated Bovine asprosin antibody is added and binds to asprosin in the sample. Subsequently, streptavidin‐HRP is added and binds to the biotinylated asprosin antibody. After incubation, unbound streptavidin‐HRP is washed away during a washing step. Substrate solution is then added and colour develops in proportion to the amount of Bovine asprosin. The reaction is terminated by the addition of acidic stop solution, and absorbance is measured at 450 nm. The detection limit of asprosin was between 0.38 and 24 ng/mL, and the functional sensitivity is 0.21 ng/mL.

### 2.5 Body Condition Score and Musculus Longissimus Dorsi Thickness

After estimation of body condition score, longissimus dorsi muscle (LDT) thickness was determined by ultrasonography. For each cattle, the paralumbar region was shaved and cleaned with ethanol. Measurements were performed with a real‐time B‐mode ultrasonography device (KX5200) using a 3.5 MHz back fat and loin muscle probe. After applying the ultrasound gel, the probe was placed perpendicular to the vertebral column between the transverse processes of the 3rd and 4th lumbar vertebrae, and measurements were performed (Termatzidou et al. 2020).

### 2.6 Pregnancy Exams

Pregnancy was diagnosed by measuring the PAG2 level from the blood samples taken on the 25 ± 2 days of the study, according to the previously described method (Fricke et al. 2016). On the 45 ± 2 days of the study, all cows were subjected to transrectal pregnancy examination using USG.

### 2.7 Statistical Analysis

All statistical analyses were performed using the R Statistical Language (version 4.1.2; The R Foundation for Statistical Computing, Vienna, Austria; https://www.r‐project.org). Shapiro–Wilk test and Q–Q plots were used to check the normality of the data. Levene test was used to assess homogeneity of variances. Variables were described as mean ± standard deviation, number (*n*) and percentage (%). Whether there was a statistically significant difference between oxidative stress, PAG2 and body condition score (BCS) findings in the LA and HA groups was determined by independent sample *t*‐test. On the other hand, the statistical difference between oxidative stress, PAG2 and BCS findings on day 0 and day 25 was evaluated with the dependent sample t‐test. Pregnancy rates of cows were compared with Fisher's exact chi‐square test in the HA and LA groups. In addition, the performance of TOS level in distinguishing cows with HA levels was investigated with the help of the ROC. The area under the ROC curve (AUC) was calculated with a 95% confidence interval, and its significance was evaluated. The most appropriate cut‐off points to distinguish HA levels of TOS were determined based on the Youden index criterion. For this determined cut‐off point, sensitivity, specificity, negative and positive predictive values were calculated with 95% confidence intervals. In evaluating two‐way hypotheses, the significance level was taken as 5%.

## 3 Results

Asprosin level on day 0 was significantly higher in the HA group compared to the LA group (*p* < 0.001). TAS levels on day 0 and day 25 were similar in the LA and HA groups (*p* = 0.797). In the HA group, the TAS level on day 25 was higher than the TAS level at the beginning of the study (*p* = 0.007). While the 25th day TOS level was similar in the LA and HA groups, the 0th day TOS level was higher in the HA group than in the LA group (*p* = 0.017). In addition, while the 0th day and 25th day TOS levels of the cows in the HA group were similar (*p* = 0.099), the 25th day TOS level of the cows in the LA group was significantly higher than the 0th day TOS level (*p* = 0.028).

**FIGURE 1:**
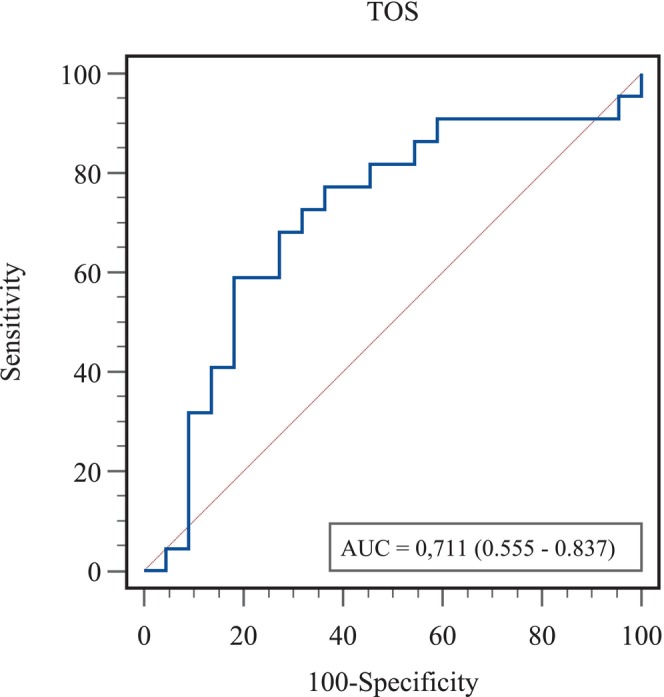
ROC Curve of TOS in distinguishing cows with high and low asprosin levels.

TOS level at the beginning of the study was higher in the HA group than in the LA group (*p* = 0.017). In conclusion, asprosin level in cows affected oxidative stress parameters. According to ROC analysis, it was concluded that there was a strong correlation between TOS and asprosin and that cows with HA values may be exposed to oxidative stress. BCS value was higher in the HA group compared to the LA group (*p* = 0.042). No difference was detected between the LA and HA groups in terms of PAG2 level, musculus longissimus dorsi thickness and pregnancy rates on the 25th day (*p* > 0.05) (Figure 1). The results are given in detail in Table 1.

**TABLE 1:** Comparison of fertility and oxidative stress parameters in cows with low (LA) and high (HA) asprosin levels.

Groups	LA (Mean ± SD)	HA (Mean ± SD)	*p*
TAS (mmol/L), day 0	0.78 ± 0.23	0.73 ± 0.09	0.340^1^
TAS (mmol/L), day 25	0.77 ± 0.14	0.83 ± 0.13	0.122^1^
*p*	0.797^2^	0.007^2^	
TOS (μmol/L), day 0	3.01 ± 1.95	4.66 ± 2.41	**0.017^1^**
TOS (μmol/L), day 25	4.58 ± 2.89	5.61 ± 2.63	0.226^1^
*p*	0.028^2^	0.099^2^	
PAG2 (ng/L), day 25	6.39 ± 2.99	5.94 ± 2.33	0.581^1^
Pregnancy (%)	82	77	> 0.999^3^
Musculus longissimus dorsi, day 0	31.46 ± 5.98	30.80 ± 6.33	0.724^1^
BCS, day 0	2.89 ± 0.26	3.08 ± 0.35	**0.042^1^**
Asprosin (ng/mL), day 0	57.62 ± 11.20	93.17 ± 23.43	< 0.001^1^
Asprosin (ng/mL), day 25	66.84 ± 30.03	62.45 ± 24.61	0.598^1^

*Note:* Data were presented as mean ± standard deviation or percentage (%). Results with statistically significant differences are indicated in bold. ^1^Independent sample t‐test; ^2^Dependent sample t‐test; ^3^Fisher’s exact chi‐square test.

The performance of TOS level in distinguishing cows with HA levels is given in Table 2. ROC analysis results showed that TOS was a statistically significant marker in distinguishing cows with HA levels from low ones (AUC = 0.711, 95% confidence interval, 0.555–0.837, *p* = 0.010). According to the Youden index, the most appropriate cut‐off point for TOS was determined as 2.67. According to this cut‐off point, the sensitivity of TOS in identifying cows with HA levels was 77.2% (95% confidence interval, 54.6%–92.2%), while its selectivity was 63.6% (95% confidence interval, 40.7%–82.8%). The positive predictive value for the TOS marker was calculated as 68% (95% confidence interval, 53.9–79.4) and the negative predictive value was 73.7% (95% confidence interval, 54.9%–86.6%) (Figure 1).

**TABLE 2:** Performance of TOS level in distinguishing cows with high asprosin levels.

	TOS
ROC analysis	
AUC (95% confidence interval)	0.711 (0.555–0.837)
*p*	0.010
Cut‐off point	> 2.67
Statistical diagnostic measures	
Sensitivity	77.2 (54.6–92.2)
Selectivity	63.6 (40.7–82.8)
Positive predictive value	68 (53.9–79.4)
Negative predictive value	73.7 (54.9–86.6)

## 4 Discussion

Based on literature information, it is predicted that asprosin level can be considered as an indicator of energy status (Romere et al. 2016; Yenilmez et al. 2024). It is also known that differences in energy status are related to oxidative stress in cows. On the other hand, both energy level and oxidative stress are known to be important factors affecting fertility success in cows (Li et al. 2022; Pedernera et al. 2010; Sordillo and Aitken 2009). For these reasons, it is predicted that differences in asprosin concentration may affect oxidative stress and fertility success in cows. Considering the above literature information, it was expected that there would be differences in the BCS score, which is an indicator of energy balance, between the groups in the presented study. When the findings of the presented study were examined, it was understood that the BCS value was higher in the HA group than in the LA group. It is thought that this is due to the fact that energy metabolism and adipose tissue lipolysis are higher in the HA group compared to the LA group. So, it was predicted that the lipolysis rate might be affected by parameter differences such as milk production between groups. However, in the presented study, such an evaluation was not made between groups. Additionally, musculus longissimus dorsi thickness was measured in this study, and no relationship was found between asprosin level and musculus longissimus dorsi thickness. Thus, although the thickness of the musculus longissimus dorsi can give an idea about the body mass index of cows, it was concluded that it did not affect the asprosin level in the presented study.

In recent studies, it is stated that asprosin is associated with oxidative stress (Wang et al. 2024; Zheng et al. 2024). A previous report reported that asprosin disrupts lipid metabolism and increases the release of ROS (Wang et al. 2024). It is thought that the relationship between asprosin level and oxidative stress may occur with the difference in energy balance between groups. Similarly, a previous study noted that energy balance was associated with oxidative stress in cows (Pedernera et al. 2010). Considering the above literature information, oxidative stress parameters are expected to be different between HA and LA groups in this study. First of all, no difference was found between the groups in terms of TAS on day 0 and day 25 in both the HA and LA groups. This can be interpreted as asprosin level does not affect the TAS level. On the other hand, it was observed that the TAS value in the LA group was similar between the 0th and 25th day measurements, while the TAS value of the HA group increased on the 25th day compared to day 0. It was assumed that this situation may be related to the TOS value. The TOS level showed a significant increase from day 0 to day 25 in the LA group. Thus, it was thought that the TAS level was used to neutralise the intensely increased TOS in the LA group. Similarly, previous studies have reported that antioxidant levels are used to neutralise oxidant substances (Ayemele et al. 2021; Sordillo and Aitken 2009).

When the TOS change between groups was examined in the presented study, the TOS level was significantly higher in the HA group than in the LA group on day 0. This has been interpreted as HA levels may increase the TOS value in cows. Similarly, a previous report stated that there was a correlation between TOS value and asprosin (Alsajri 2022). Therefore, it is thought that cows with HA values may be exposed to oxidative stress. Although there are limited studies in this field, previous studies indicate that increased asprosin value may be associated with oxidative stress (Rangraz Tabatabaei et al. 2023). It is assumed that asprosin level is directly proportional to insulin resistance and therefore may be related to oxidative stress (Feng et al. 2018). Supporting this situation, some studies reported that insulin resistance is associated with oxidative stress in cows (Abuelo et al. 2016; Wu et al. 2020). On the 25th day of the study, no significant difference was found between the LA and HA groups in terms of TOS value. Assuming that the asprosin level is affected by energy demand (Romere et al. 2016), it was thought that the energy balance between the groups was equal on the 25th day of the study, and therefore the TOS production from lipolysis was similar. However, TOS values on day 0 and day 25 were similar in the HA group. This result was thought to be related to the TAS value of the HA group. In the HA group, TAS value increased on the 25th day compared to the 0th day of the study. Thus, it was assumed that the increased TAS value prevented the increase in TOS by neutralising the currently produced oxidant substances. In the LA group, the TOS level was found to be significantly higher on the 25th day compared to the 0th day. This result cannot be interpreted clearly. However, it is estimated that the fact that the TAS level did not increase on the 25th day in the LA group caused the existing TOS production to accumulate without being neutralised. Similarly, many studies have reported that antioxidants are used to neutralise oxidant substances (Abuelo et al. 2015; Ayemele et al. 2021; Ponnampalam et al. 2022; Sordillo and Aitken 2009).

When the presented study was considered in terms of fertility parameters, no significant difference was detected in the pregnancy findings obtained by PAG2 level measurement on the 25th day of the LA and HA groups. However, when the number of pregnancies between the groups was examined, it was determined that the pregnancy rate was higher in the LA group than in the HA group. Since there was no statistical difference, it was thought that this situation was not related to asprosin level. However, previous studies have stated that asprosin level is associated with many fertility parameters such as ovarian steroidogenesis mechanism, follicle development and polycystic ovary syndrome (Batalha 2020; Batalha et al. 2023). In the presented study, no other evaluation was made as a fertility parameter other than pregnancy rate. In this context, in order to clearly determine the effect of asprosin on fertility, it is thought that parameters such as cyclic activities, ovarian health, early and late embryonic death should also be evaluated.

In the study, pregnancy diagnoses of cows were made by PAG2 measurement on the 25th day of pregnancy. This method was determined based on previous research (Barbato et al. 2022; Commun et al. 2016). However, since fertility success is critical for the study outcome, the pregnancies of cows diagnosed by PAG2 analysis were confirmed by transrectal USG on the 45 ± 2 days of the study. On the other hand, cows diagnosed with pregnancy in the study successfully completed the pregnancy and gave parturition. Similarly, according to the PAG2 result, non‐pregnant cows were subjected to USG examination on the 45 ± 2 days of the study. These findings confirmed the fertility status diagnosed based on the PAG2 result in the study. Therefore, the fertility results in the presented study are fully accurate. Although the direct relationship of asprosin on PAG2 value is not stated, it is thought that asprosin level may affect the PAG2 level due to its effect on oxidative stress (Ayad et al. 2020; Merlob et al. 2008). However, in this study, it was determined that the PAG2 value was similar between the groups on the 25th day. Additionally, it was evaluated by ROC analysis whether cows with high and low asprosin (LA) levels could be identified according to other parameters. In the presented study, ROC analysis results revealed that TOS was an effective marker in distinguishing cows with HA levels from those with low levels. According to this result, it is predicted that the TOS value is correlated with the asprosin level and that these values can be used as an indirect prediction tool in determining the asprosin level.

## 5 Conclusions

In conclusion, asprosin level affects oxidative stress parameters in cows. The direct effect of asprosin on antioxidants could not be clearly expressed. However, it was determined that its effect on TOS was clearer. When this situation was evaluated by ROC analysis, it was concluded that there was a strong correlation between TOS and asprosin and that cows with HA values may be exposed to oxidative stress. Asprosin level had no effect on PAG2 and pregnancy parameters. It is thought that this situation should be investigated in more detail in future studies. Additionally, in future studies, the relationships between asprosin value and ovarian health, cyclic activity and embryonic deaths should be investigated. It is recommended to use larger groups of cows for this purpose.

## Author Contributions

**Rahmi Köse:** investigation, data curation, writing – original draft. **Serkan Ali Akarsu:** funding acquisition, project administration, writing – review and editing. **Serdal Kurt:** formal analysis, writing – review and editing. **Hakan Serin:** statistical analysis. **Mehmet Akif Aydın:** data curation. **Ahmet Karahanlı:** methodology. **Gamze Uçak:** investigation, formal analysis. **Ahmet Yörü:** investigation.

## Conflicts of Interest

The authors declare no conflicts of interest.

## Data Availability

The data that support the findings of this study are available from the corresponding author upon reasonable request.
